# One Side of the Story; Clues to Etiology in Patients with Asymmetric Chorea

**DOI:** 10.5334/tohm.675

**Published:** 2022-01-31

**Authors:** Molly Cincotta, Ruth H. Walker

**Affiliations:** 1 University of Pennsylvania; 2 Parkinson’s Disease and Movement Disorder Center, US; 3 Department of Neurology, James J. Peters Veterans Affairs Medical Center, US; 4 Department of Neurology, Mount Sinai School of Medicine, US

**Keywords:** chorea, unilateral, asymmetric, hemiballismus, hemichorea

## Abstract

**Background:**

Chorea can be due to a large number of etiologies. Unilateral chorea is classically related to a contralateral structural lesion, e.g. of the putamen or subthalamic nucleus, however, based upon personal impressions, we have observed that systemic disease, in particular metabolic or autoimmune conditions, can also lead to a unilateral or markedly asymmetric presentations. We sought to investigate this impression by reviewing the literature.

**Methods:**

A PubMed search was conducted using the terms asymmetric” AND “chorea” OR “hemichorea” OR “unilateral” AND “chorea” OR “monochorea” OR “right greater than left” AND “chorea” OR “left greater than right” AND “chorea” OR “right more than left” AND “chorea” OR “left more than right” AND “chorea” as well as “hemiballismus” NOT “stroke” NOT “infarct” NOT “dyskinesia. A total of 243 sources were felt to meet criteria and were reviewed.

**Results:**

The most common etiology of reported hemi- or asymmetric chorea was diabetic non-ketotic hyperglycemic hemichorea/hemiballismus. Other common diagnoses were Sydenham’s disease, antiphospholipid syndrome and drug-induced chorea. The vast majority of patients with hemi- or asymmetric chorea had acquired rather than genetic, degenerative or congenital causes.

**Conclusion:**

Despite the potential limitations of our literature review, the evidence presented here supports the observation that the vast majority of asymmetric or unilateral chorea presentations are due to acquired causes, and in this situation an exhaustive search for reversible etiology should be undertaken. However, presentation with symmetric, generalized chorea does not exclude reversible causes, and investigations should address these in addition to genetic and neurodegenerative etiologies.

## Introduction

Chorea is a hyperkinetic movement disorder described as random, flowing, and dancelike [1]. In many conditions chorea is generalized, involving the entire body, however, in a significant number of situations, the movement disorder can be strikingly asymmetric or even unilateral.

While unilateral involvement is classically related to a contralateral structural lesion, e.g. of the putamen or the subthalamic nucleus, with stroke as the most common etiology, systemic disease can also lead to a unilateral or markedly asymmetric presentations [1]. In particular, hyperglycemic hemichorea/hemiballismus is typically described as being unilateral with transient T1 hyper intensity of the contralateral putamen on magnetic resonance imaging [2,3,4,5]. Additionally, the commonest cause of childhood chorea, Sydenham’s disease, is commonly asymmetric, even when generalized [6,7,8]. The mechanism underlying of the asymmetry in these systemic disorders is not well understood.

In contrast, in the genetic choreas the movement disorder is typically midline or symmetric in presentation, although sometimes with a particular anatomic predilection. In particular, Huntington’s disease, the most common cause of genetic chorea, is one of the few disorders specifically noted to have forehead chorea as a potentially diagnostic characteristic [1]. In other disorders, such as in patients with hexanucleotide repeat expansions in *C9ORF72* with chorea or chorea-acanthocytosis, there is involvement predominantly of the lower face and tongue [9,10].

As many acquired forms of chorea may result from underlying disorders that require specific targeted treatment, early identification is critical. In addition, understanding potential etiologies for asymmetric chorea presentations might help to unify some of the pathophysiologies behind these conditions.

In this review, we endeavor to summarize the literature on asymmetric chorea as relates to the underlying diagnosis to support this observation and to clarify this topic.

### Literature review

We searched the PubMed/MEDLINE database for relevant literature in June of 2021. Search terms included “asymmetric” AND “chorea” OR “hemichorea” OR “unilateral” AND “chorea” OR “monochorea” OR “right greater than left” AND “chorea” OR “left greater than right” AND “chorea” OR “right more than left” AND “chorea” OR “left more than right” AND “chorea” (Supplemental Figure 1). 886 items were identified. Papers not written in English or without English translations were excluded, 49 in total. Of the remaining documents abstracts were reviewed and documents not pertaining to the search topic were excluded as were general reviews, animal studies and cases where structural lesions were clearly the cause of the hemichorea. Of the remaining documents an additional 57 were excluded on review of the full document due to recognition of the above criteria. Other causes for exclusion included chorioathetoid movements secondary to cerebral palsy or birth injury, trauma, vasculopathy-induced hemichorea even in the absence of stroke and levodopa-induced dyskinesia in idiopathic Parkinson’s disease. Papers for which access to the full text was not accessible through the primary institution were also excluded. Following this initial review an additional search was conducted on November 3, 2021 for “hemiballismus” with exclusion of the terms “hemichorea,” “stroke,” “infarct” and “dyskinesia.” This yielded 41 English language results which were then narrowed to 8 after excluding non-relevant papers as described above.

The remaining 229 documents were reviewed in full. An additional 6 sources were added due to relevance despite not being identified during the search. This total of 243 sources was further subdivided based on broad category of diagnosis (e.g. metabolic or inflammatory), specific diagnosis (e.g. hyperglycemic or Sydenham), MRI findings related to the presentation, presence of any abnormal advanced imaging or pathological analysis, and the authors’ conjectures as to the cause of asymmetry (Supplemental Figure 2).

## Results

### Metabolic causes

#### Hyperglycemic hemichorea

The first case of diabetic non-ketotic hyperglycemic hemichorea/hemiballism (HC/HB) was described by Rector in 1982, who published three cases of unilateral chorea in patients with non-ketotic hyperglycemia whose movements resolved shortly after correction of their metabolic imbalance [11]. Since that time, non-ketotic HC/HB has been recognized as the second most common cause of hemichorea after acute infarct [4]. This was borne out in our literature search with 153 distinct references describing this diagnosis. This condition typically presents as an acute to subacute hemibody chorea in an elderly patient with non-ketotic hyperglycemia. Women appear to be more susceptible than men [3,12,13,14]. Many of the initial cases were described in those of East Asian descent, although subsequent reviews have not consistently supported a specific ethnic susceptibility [3,13]. Classic imaging demonstrates contralateral basal ganglia (putamen more than caudate) hyperintensity on T1 MRI sequencing and hyperdensity on CT in a similar region, although normal imaging is also common [3,5,12,13,14].

Many variations of this presentation have been described, including bilateral MRI changes [15,16,17,18,19,20,21,22,23,24], ipsilateral MRI changes [25], bilateral chorea [23,26], recurrent chorea [27,28] and persistent chorea [25,29,30]. Cases have been described in the pediatric population [31,32,33,34,35,36] as well as in both children and adults presenting with ketotic hyperglycemia [22,37,38,39]. (Please see supplementary Figure 3 for a summary of imaging findings in hyperglycemic HC/HB.)

The underlying pathophysiology of hyperglycemic HC/HB is not completely understood, although many theories have been proposed including metabolism of gamma-aminobutyric acid (GABA) as an alternate energy source, ischemic injury, deposition of metalloproteins, hemorrhagic injury, derangements in dopamine or estrogen function, autoimmune, anti-glutamic acid decarboxylase (anti-GAD)-mediated autoimmunity or neurodegeneration of myelinated axons [40]. Although most of these conjectures have merits, only some address the issue of the striking asymmetry observed in most cases.

Hypotheses regarding the cause(s) of hyperglycemic HC/HB are often based upon the very striking imaging, as few other pathologies result in increased density on both CT and the T1 sequence of MRI. The most common early explanation was that HC/HB was secondary to microhemorrhages, however autopsy data did not entirely bear this out [2,41]. Neuropathological examination demonstrated chronic infarctions with punctate calcifications and mineralizations, in addition to swollen axons and gemistocytic astrocystosis [41]. The gemistocytes have been theorized to be the cause of the T1 hyperintensity, as has potential formation of manganese superoxide dismutase from oxidative stress [41]. MR spectroscopy showed decreased NAA/Cr ratio and increased Cho/Cr ratio, suggestive of obliterative vasculopathy with prominent vascular proliferation, which would be consistent with a microischemic etiology for the syndrome [42]. Chronic ischemic damage might also account for a case of prolonged chorea and caudate atrophy in a patient with untreated hyperglycemic HC/HB [30].

The uniting theory, therefore, would be that asymmetric microvasculopathy, likely driven by uncontrolled diabetes mellitus and other risk factors, would create vulnerability to metabolic insult in the setting of extreme hyperglycemia. While large vessel ischemic vulnerability seems to be a rare contributing factor, one report noted middle cerebral artery (MCA) stenosis ipsilateral to the MRI lesion [43]. While it is not possible to know whether this was an incidental finding, this observation would support a unilateral perfusional vulnerability. In this model, CT and MRI changes would be secondary to swollen astrocytes, gemistocytes and mineral deposition due to oxidative stress from metabolic dysregulation. This may also be consistent with the most common pathophysiological conjecture that suggests while in a state of anaerobic metabolism, the highly metabolically active striatum and subthalamic nucleus convert from glycolysis to use of GABA as an energy source. The subsequent dearth of inhibitory neurotransmitter disrupts the thalamic-cortical feedback loop resulting in the hyperkinetic movements [40]. A pre-existing vascular asymmetry, either anatomical or pathologic, could possibly account for increased vulnerability of the basal ganglia on one side in the majority of patients.

Functional imaging has provided contradictory data. Nine sources examined patients with [^18^F]-fluorodeoxyglucose (FDG) positron emission tomography (PET) scans. Seven of these demonstrated decreased glucose metabolism of the contralateral basal ganglia, often corresponding with the region of T1 hyperintensity on MRI [17,28,42,44,45,46,47]. One case series imaged two patients at different time points; at nine days after symptom onset there was a contralateral increase in glucose metabolism, while at fifty-five days after onset there was a relative decrease [39]. Of note these patients were both presenting with ketotic (rather than non-ketotic) hyperglycemia. Another atypical patient, with hyperglycemic hemichorea contralateral to bradykinesia from parkinsonism, also showed increased glucose uptake in the bilateral basal ganglia, most marked contralateral to her hyperkinetic movements [39]. Overall these examinations would suggest fluctuations in glucose metabolism in the setting of hyperglycemia, with decreased uptake being the most common finding in typical cases. There is some thought that the variability might be due to the timing of scans—i.e. scans done earlier might show increased metabolism and scans done later might show decreased or vice versa—however one study that looked at three patients five days, 10 days and 1 month respectively all noted decreased glucose metabolism, arguing against this idea [45].

The same study [45] also examined hexamethylpropyleneamine oxime (HMPO) single photon emission computerized tomography (SPECT) scans in two patients, demonstrating increased perfusion of the contralateral basal ganglia. This finding was consistent with easy Z-score (eZis)-assisted SPECT findings and [123] I-N-isopropyl-p-iodoamphetamine SPECT, which also demonstrated increased blood flow to the contralateral striatum and thalamus [45,48]. Combined with the pathological data and FDG-PET imaging, this could perhaps suggest that the decrease in glucose metabolism combined with ischemia drives an increase in perfusion, as opposed to increased activity drawing increased blood flow due to neurotransmitter feedback disruption.

Dopamine transporter (DaT) SPECT imaging in two case reports demonstrated decreased uptake contralateral to the affected side [49,50]. In both cases the patients did not demonstrate clinical parkinsonism and no follow up was reoirted to determine whether these patients went on to develop PD or if the DaT changes remained with resolution of their chorea [49,50]. One case speculated that the finding was clinically significant and represented true decreased presynaptic dopaminergic uptake [49] while the second suggested that the finding was secondary to structural changes in the basal ganglia related to the T1 changes on MRI [50]. The bases for these speculations are unclear, and, in the absence of supporting evidence and more systemic studies, it would appear more likely that these patients had presymptomatic PD. In a patient who initially presented with persistent hyperglycemic HC/HB and later developed contralateral parkinsonism, DaT SPECT showed bilateral decreased uptake despite unilateral parkinsonian symptoms [51].

It should be noted that both hyper- and hypo-glycemia can lead to focal neurologic deficits as well as focal seizure activity that could be mistaken for abnormal movements. One case of hyperglycemic HC/HB was reported to have periodic lateralized epileptiform discharges (PLEDs) in associated with persistent symptoms, albeit refractory to anti-epileptic medications [52]. Focal and generalized seizures are reported in multiple cases of hyperglycemic HC/HB [53,54,55]. This, along with imaging showing delayed gadolinium enhancement, has led to conjecture that breakdown of the blood-brain barrier secondary to hyperglycemia might contribute to the pathogenesis of both neurologic manifestations [55,56].

Another proposed mechanism for asymmetry in hyperglycemic HC/HB is hyperviscosity [57]. This will be discussed more thoroughly in the section on polycythemia vera as this is another established caused of hemichorea.

#### Hypoglycemia

Hypoglycemia has also been implicated as a cause of hemichorea. One report described one patient with generalized chorea and another with hemichorea [58]. Both patients had lost consciousness with blood glucose levels below 50 milligrams/deciliter. Both patient had MRI scans that showed T2 hyperintensity of the bilateral basal ganglia, and one had a SPECT scan demonstrating hyperperfusion in the same region. Neither patient had reported hyperglycemia preceding the event [58].

In other case reports hypoglycemia at presentation was preceded by elevated hemoglobin A1C levels [59], or by a recent episode for non-ketotic hyperglycemia [60]. This raises the possibility that the abnormal movements were a delayed response to hyperglycemia, and that the hypoglycemia at presentation was incidental. This was supported by imaging demonstrating the classic basal ganglia T1 hyperintensities and hypometabolism on FDG-PET, as seen in hyperglycemic HC/HB [59,60].

#### Disorders of Heavy Metal Metabolism

There are some cases of heavy metal deposition, whether genetic or acquired, resulting in asymmetric chorea. While symptoms were asymmetric, in some conditions there was no appreciable asymmetry on imaging. In one case of asymmetric chorea associated with hypermanganesemia from chronic high tea consumption, MRI of the brain showed fairly symmetric bilateral manganese deposits in the basal ganglia. Both imaging and symptoms improved after chelation [61].

Asymmetric chorea was reported in Wilson’s disease [62,63] and aceruloplasminemia [64]. In the Wilson’s cases, MRI lesions corresponded with the affected side, however in the aceruloplaminemia case the changes were symmetric. Another case described late-onset hemiballismus in a patient with neurodegeneration with brain iron accumulation type 1 without a clear lesion on autopsy [65].

#### Vitamin/Mineral Imbalance

In additions to derangements of glucose, other metabolic abnormalities can cause asymmetric chorea in the setting of a pre-existing structural lesion. A 5-year-old boy with a prior right-hemispheric stroke developed left-sided chorea when he developed vitamin D deficiency leading to hypocalcemia and hypomagnesemia [61]. Several cases of hyperglycemic hemichorea also followed this pattern, where previous focal lesion or asymmetric white matter injury seemed to predispose to unilateral basal ganglia overactivity [61,66,67]. Hypoparathyroidism has been associated with hemiballismus, but this was in the setting of extensive calcifications of the basal ganglia which, while grossly symmetric, were assumed to be the true underlying cause for the abnormal movements [68].

#### Uremia

Uremic chorea is an entity with some potential overlap with hyperglycemic hemichorea. In one review of 15 patients, the main differences as compared with non-ketotic hyperglycemia, beyond the serum metabolic panels, were the lack of T1 hyperintensities in the uremic patients. Both diseases shared T2 hyperintensities in the basal ganglia, however in the uremic patients these lesions were more edematous and had a rim of strong T2 hyperintensity not seen in the hyperglycemic patients. There also appeared to be a larger proportion of patients presenting with generalized (presumably symmetric) versus asymmetric chorea in the uremic patients compared to the hyperglycemia patients, albeit with a small sample size [69].

#### Genetic metabolic derangement

Hemichorea due to metabolic abnormalities of genetic etiology was reported in the case of a six-year-old girl who presented with language regression, hemichorea and focal seizures, who was ultimately found to have a GLUT-1 deficiency. As this disease affects glucose transport across the brain-brain barrier, and the patient was found to have hypoglycorrhachia, one might conjecture a similar pathogenesis to that of acquired hypoglycemia. Imaging showed a hyperintense, linear lesion of the left external capsule on T2 fluid-attenuated inversion recovery [70]. Asymmetry of hyperkinetic movements has not been noted in other patients with this condition.

### Automimmune Inflammatory casyes

#### Sydenham’s disease

Sydenham’s disease is the most common cause of acquired chorea in children, and often presents asymmetrically or unilaterally [8,71]. Although there are few cases currently seen in resource-rich countries, it remains quite prevalent in many resource-limited countries [71]. Although known for its asymmetry, in one follow-up study of 65 children, 78% were reported as generalized versus 22% hemichorea. It was unclear from the paper if some of these generalized cases were asymmetric [8].

The etiology of Sydenham’s disease is proposed to be an autoimmune response generated by antibodies to streptococcus A, often in the setting of rheumatic fever. Onset of symptoms is generally 1–8 months after the acute infection, although cases are reported of abnormal movements as the presenting symptom [71,72].

MRI is typically normal or nonspecific, although there is a report of basal ganglia edema and subsequent cystic atrophy in one three-year-old patient [73]. SPECT has demonstrated reversible contralateral striatal hypermetabolism in patients with strictly unilateral hemichorea [74]. Perfusion SPECT studies have shown hyperperfusion of the contralateral basal ganglia in most cases of both generalized and asymmetric chorea, although normal perfusion or hypoperfusion was noted in some cases [74,75].

Sydenham’s disease has been conjectured to be similar to other infectious/inflammatory processes that result in vasculitis with destruction of the blood-brain barrier, exposing the basal ganglia to anti-neuronal antibodies formed in molecular mimicry to streptococcal antigens [75,76]. The hyperperfusion, therefore, would be secondary to overactivity of the basal ganglia when exposed to these antibodies [75]. Asymmetry could be secondary to differential vascular effects of the inflammation and a differing degrees of blood brain barrier breakdown. However, pathological evaluations at the time of the movements are lacking.

It is notable that patients who have a history of Sydenham’s disease go on to have higher rates of chorea gravidarum. This diagnosis will be discussed in more detail in a later section, however one study that specifically found that 15 of 20 patients (75%) who became pregnant after a history of Sydenham’s disease developed chorea gravidarum [77]. This suggests that, despite the apparent remission of this symptom, there may be more permanent structural or neurochemical vulnerabilities that are not detected by conventional imaging.

#### Paraneoplastic/Autoimmune Encephalitides

Paraneoplastic or autoimmune encephalitis is characterized by auto-antibodies that target neuronal tissue and can cause a spectrum of neuropsychiatric symptoms, including encephalopathy, psychosis, seizures and abnormal movements. The best described of these is anti-N-methyl-D-Aspartate (NMDA) receptor encephalitis, which results in characteristic facial dyskinesia as well as other hyperkinetic movements such as hemichorea [78,79,80]. Although hemichorea is not usually the only presenting symptom, cases have been reported with movement disorders, including hemichorea, preceding the more recognizable psychiatric or cognitive symptoms [80].

The most common cause of paraneoplastic chorea is anti-collapsin response-mediator protein 5 (anti-CRMP-5; anti-CV2) immunoglobulin (Ig) G. Hemichorea has been noted in case reports of patients with small cell lung cancer [81] and diffuse large B-cell lymphoma [82]. Imaging in the former case was described as showing bilateral T2 hyperintensities of the basal ganglia, although the latter reported normal MRI imaging. A review of 16 cases noted that the majority presented with generalized chorea, often with T2 hyperintensities in the bilateral basal ganglia and mesial temporal lobes [83].

Anti-leucine-rich, glioma inactivated-1 (LGI-1) antibodies typically cause facio-brachial dystonic seizures, however chorea has also been reported. The chorea has been described as generalized and asymmetric, often starting in one area of the body and subsequently spreading to involve other regions [84,85,86]. This condition is typically not associated with a neoplasm, although in one case it was suspected to be related to renal cell carcinoma [85]; all symptoms appear to be exquisitely sensitive to immunotherapy.

#### Anti-phospholipid Antibody Syndrome (APS) and Systemic Lupus Erythematousus (SLE)

APS with or without SLE has been noted as a cause of asymmetric chorea. This symptom appears to generally occur in the absence of acute infarct in a region that would be reasonably associated with hyperkinetic movements, and thus has been presumed to be a direct autoimmune phenomenon in these patients [87]. This is also reinforced by a case report of a patient presenting with chorea who had high antiphospholipid antibody titers but no clinical APS, suggesting the antibodies themselves induce the movement [88]. Like Sydenham’s disease, APS chorea is more common in children and appears to be estrogen-sensitive, occurring more frequently in female patients and occurring or recurring during exposure to oral contraceptives and pregnancy [87,89]. FDG-PET imaging has demonstrated hypermetabolism of the contralateral basal ganglia [90,91].

Chorea can occur in APS with or without association with SLE [90,92]. Although SLE can independently cause chorea [93] it is more frequently associated with positive antiphospholipid titers, even if the patient previously tested negative for APS [94].

#### Other Autoimmune Inflammatory causes

Other reports of autoimmune/inflammatory causes of chorea include Churg-Strauss [95], celiac disease [96], myasthenia gravis [97], and lymphocytic hypophysitis [98]. Of note, there is also case report of a patient with a pituitary macroadenoma causing hemichorea, but this was in the setting of secondary hyperglycemia and thus the chorea was felt to be similar to that of hyperglycemic HC/HB rather than direct effect of the pituitary enlargement [99]. Grave’s disease has also been implicated as a cause of hemichorea, but this is likely secondary to hyperthyroidism and will be discussed further in the endocrine section [100].

### Infectious causes

Infectious causes are not a common cause of hemichorea when not associated with a clear structural lesion, as can be seen in toxoplasmosis. Neurosyphilis and salmonella typhi have both been reported to cause chorea in single case reports, in which there was no structural lesion but symptoms were asymmetric [101,102]. One pediatric case of 2-year old who developed hemiballismus secondary to Influenza A infection was also reported. Notably in that case there were reversible unilateral imaging changes on MRI and the etiology was ultimately attributed to acute necrotizing encephalopathy [103]. HSV encephalitis with hemichorea demonstrated bilateral striatal MRI changes, but the presentation was felt to be due to direct infection [68].

### Hormone Imbalance

#### Chorea gravidarum/Estrogen-induced chorea

Chorea gravidarum is a rare but well-established hemichorea that occurs during pregnancy, typically appearing after the first trimester and resolving after delivery [104]. As previously discussed, there is a strong association to a history of inflammatory chorea such as Sydenham’s disease or APS [77,104].

The underlying cause of chorea gravidarum appears to be increased levels of estrogen; patients who experience abnormal movements during pregnancy are at increased risk of hemichorea in association with oral estrogen, whether from oral contraceptive pills containing estrogen or from post-menopausal hormone replacement therapy [105,106,107,108] Studies have shown that estrogen increases dopamine release in the brain through several different mechanisms as well as by increasing dopamine receptor density post-synaptically [104]. The mechanism for estrogen-induced hemichorea, whether through pregnancy or oral administration, thus appears to be on the basis of pre-existing sensitivity. PET studies have shown contralateral glucose hypermetabolism in the basal ganglia, focused around the caudate nucleus [109], which is thus consistent with increased dopaminergic activity.

#### Hyperthyroidism

The other most common cause of hemichorea from an endocrine source unrelated to diabetes, is hyperthyroidism. Cases have been described both with symmetric and asymmetric chorea [100]. Movements resolve with treatment and tend not to recur, [100,110,111] unless treatment is discontinued [111].

The etiology of hyperthyroid-induced chorea is unclear but is theorized to be due to neurotransmitter dysfunction, including noradrenergic, serotonergic and cholinergic systems, as well as increasing the sensitivity of the basal ganglia dopamine receptors [100,111]. Additionally, there have been cases of Graves’ disease associated with reversible vasculitis, although this was not the case in any of the reported hemichorea cases and therefore this seems unlikely to be the cause of the abnormal movements in this condition [112]. A direct effect of thyroid hormone or anti-thyroglobulin antibodies on the basal ganglia has also been proposed [100].

### Medications/Toxic exposure

The most common cause of drug-induced asymmetric chorea is secondary to levodopa use in patients with idiopathic Parkinson’s disease who develop dyskinesia, however, this is outside of the scope of this review. Multiple other medications have been identified as causing an asymmetric or hemichorea including gabapentin [113], valproate [114], amphetamine [115], anti-histamine [116,117], selective serotonin reuptake inhibitors [118], neuroleptics [119] and z-drugs [120]. As previously mentioned, oral estrogen has been associated with drug-induced chorea as well, although this appears to be through a specific hormonal mechanism [105,106,107,108,109]. Medication-induced chorea appears to be a heterogeneous group of conditions, likely with multiple mechanisms of action. The etiology of the asymmetry is unclear, although case reports of both symmetric and asymmetric presentations have been noted, suggesting that in some cases there may be underlying brain pathology or vascular changes that predispose to unilateral basal ganglia involvement [114,119].

Carbon monoxide generally causes symmetric injury to the basal ganglia and can cause acute or delayed neurological effects, although chorea is rare. In one reported case, with chorea isolated to one lower limb, MRI changes appeared fairly symmetric without focal basal ganglia lesions [121].

### Polycythemia vera

Chorea is a well-described but rare complication of polycythemia vera, a myeloproliferative disorder associated with a genetic mutation of *JAK2V617F* and is classically unilateral or highly asymmetric [122,123,124,125]. The etiology is felt to be secondary to hyperviscosity leading to venous stasis, impaired oxygenation and deranged glucose metabolism [124]. Slower blood flow is postulated to alterations in turnover of cerebral catecholamines and serotonin, resulting in receptor up-regulation [125]. Hyperviscosity as an etiology of hemichorea might also link the pathophysiology of polycythemia vera chorea to hyperglycemia HC/HB [57]. Imaging in polycythemia vera may be normal or show symmetric or asymmetric T2 basal ganglia hyperintensities [122,125]. Although stroke should be considered in any case of sudden onset hemichorea, especially in a predisposing condition like polycythemia, acute infarct is not a clear cause of this presentation [124].

### Genetic etiology

Although genetic causes of asymmetric chorea are not common, they are occasionally reported. One case report included an infant with sudden onset of choreoathetoid movements of the right side of his body after a febrile illness who was later diagnosed with glutaric aciduria type I [126]. MRI showed slight increased T2 signal in the bilateral basal ganglia, especially in the head of the caudate nucleus, more prominent on the left.

### Neurodegenerative etiology

With respect to neurodegenerative disorders, excluding levodopa-induced dyskinesia as mentioned above, there was a single case report of alternating hemichorea in a patient with progressive supranuclear palsy [127]. Despite numerous reports of asymmetric caudate atrophy in Huntington’s disease, there is only one case report which mentions a patient with asymmetric chorea and this was in the setting of treatment with multiple neuroleptics for presumed schizophrenia [128].

### Other causes

One case report involved migraine with aura and right-sided chorea in a 57-year-old woman with established migraine history. Perfusion imaging demonstrated asymmetric decreased blood flow to the left subthalamic nucleus which resolved on repeat imaging when the patient was asymptomatic [129]. There was also decreased perfusion to the left occipital lobe. The authors drew connections between the increased stroke risk in migraine associated with increased exposure to anticardiolipin or antiphospholipid antibodies and the chorea of antiphospholipid syndrome, although the patient tested negative for these antibodies and chorea is a rare manifestation of migraine.

Post-pump chorea is a rare complication of cardiac surgery in the pediatric, and occasionally adult, population and is usually generalized. A case of post-pump hemichorea was reported in a 9-year-old girl after aortic membrane resection performed on cardiopulmonary bypass [129]. Imaging in this case was unremarkable.

## Discussion

In this review we found that the vast majority of cases of asymmetric or hemichorea unrelated to structural lesions were due to acquired conditions, including, but not limited to, hyperglycemic HC/HB, Sydenham’s and other autoimmune diseases, polycythemia vera, and chorea gravidarum.

However, with the exception of some specific disorders, such as hyperglycemic HC/HB and chorea gravidarum/estrogen-induced hemichorea which are typically asymmetric or unilateral, chorea of metabolic/autoimmune etiology can be either symmetric or asymmetric.

The explanation for the asymmetric phenomenology is not apparent, as many of the conditions discussed here are systemic and would therefore be expected to cause bilateral basal ganglia dysfunction. The reason for an asymmetrical presentation for most of these cases is unclear, but some hypotheses can be generated.

One uniting theme is that of asymmetric blood flow and microscopic ischemic injury increasing vulnerability to systemic insult. In the case of hyperglycemic HC/HB there are clear macroscopic cases, such as pre-existing severe large vessel stenosis [130] or stroke, [66,67]. In most cases, however, the changes appear to be chronic and microscopic with suggestion of long term injury on pathology [41,42] that is not readily seen by MRI and therefore is hard to prove. In the case of inflammatory disorders, the picture is even more muddied, as many disorders can have multiple types of nervous system involvement, so causality is not always clear. In diseases such as SLE or Churg-Strauss, some degree of vascular inflammation may be expected, however pathological specimens are lacking and imaging changes are inconsistent [94,95]. It has also been proposed that ischemic changes might in fact *suppress* an otherwise generalized chorea in cases with ipsilateral lesions [94]. Vasculitis has been noted as a potential cause of the breakdown of the blood brain barrier in Sydenham’s disease, increasing vulnerability to circulating chorea-inducing autoantibodies [76], although again direct evidence for this in the form of pathology or consistent imaging could not be found.

Another theme of our review is that of hyperviscosity, in the setting of medium and large blood vessel asymmetry. This can be seen most obviously in circumstances such as polycythemia vera, where slow flow (“sludging”) secondary to high hematocrit is considered the primary explanation for hemichorea [122]. APS could result in a similar pathophysiology, as it is a disorder of hypercoagulability, although this would not explain cases where there were high antibody titers without clinical APS [88]. Hyperglycemic HC/HB has also been conjectured to be secondary to hyperviscosity. Brain regions with pre-existing compromised blood flow on the basis of structural vascular asymmetry could certainly be hypothesized to be more vulnerable in hypercoagulable conditions.

One alternative explanation may be an intrinsic asymmetry in basal ganglia structures and pathways [110], as seen, for example, in the typically asymmetric presentation of Parkinson’s disease. An asymmetric dopaminergic input to the basal ganglia, or a subtle structural asymmetry of one of the other nuclei, might result in imbalance between the direct and indirect pathways, and increased activity of the latter unilaterally resulting in hemichorea.

As most of the conditions described here are reversible, relatively few come to autopsy, and any imaging abnormalities tend to be temporary. Further functional imaging studies may be the most rewarding path to pursue in elucidating the mechanisms underlying these disorders; these tend to show basal ganglia hypermetabolism, which may be another diagnostic clue [131].

### Limitations

Our review is limited by the variable terminology and levels of description in the literature. Although some of the larger case series and retrospective studies noted asymmetric cases that were not unilateral, most divided patient presentations into hemichorea versus generalized chorea. Some of the case reports that did not appear on initial search but were identified subsequently had reference to asymmetry mentioned in passing within the body of the text. As such it is possible that degenerative cases were overlooked because the authors either failed to find the asymmetric presentation notable enough to include or because the search terms did not identify a description within the article text. Also, as mentioned above, many of the asymmetric cases were written and published because of their notability and might underrepresent the presence of symmetric generalized chorea as an alternate presentation of that disorder.

## Conclusion

Despite these limitations, the evidence presented here indicates that the vast majority of asymmetric or unilateral chorea presentations are due to acquired causes, and in this situation an exhaustive search for reversible etiology should be undertaken. However, conversely, presentation with symmetric, generalized chorea does not exclude reversible causes, and investigations should address these in addition genetic and neurodegenerative causes.

## Additional Files

The additional files for this article can be found as follows:

10.5334/tohm.675.s1 Supplemental Figure 1. Results of literature search.

10.5334/tohm.675.s2 Supplemental Figure 2. Number of papers by diagnosis.

10.5334/tohm.675.s3 Supplemental Figure 3. Imaging findings in hyperglycemic hemichorea/hemibalismus.
