# Supplementary figures and images for: One Side of the Story; Clues to Etiology in Patients with Asymmetric Chorea
Source: Tremor Other Hyperkinet Mov (N Y). 2022 Jan 31;12:3. doi: 10.5334/tohm.675 (PMC8815437; doi:10.5334/tohm.675)

Supplemental Figure 1: Results of literature search.

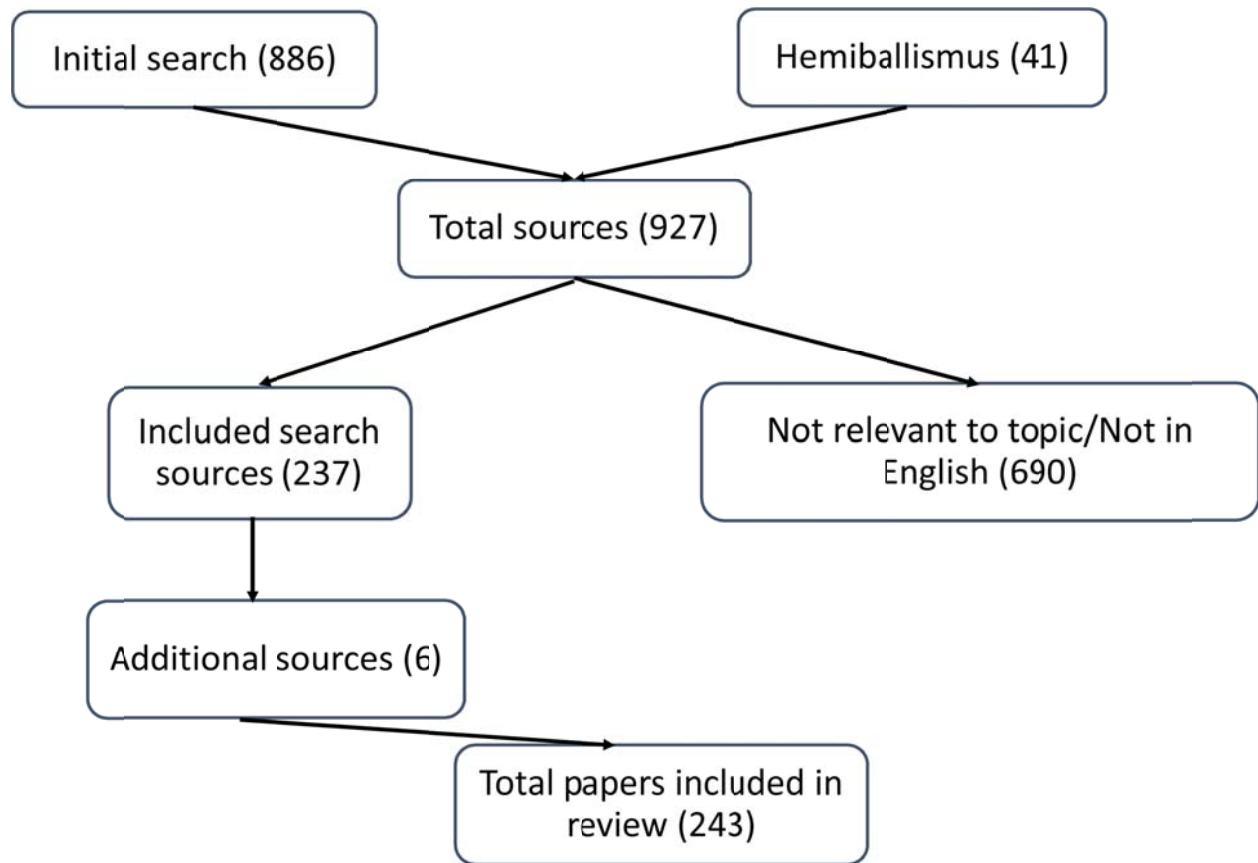

Supplement: Supplemental Figure 1. — Results of literature search. [file tohm-12-1-675-s1.pdf]

Supplemental Figure 3: Imaging findings in hyperglycemic hemichorea/hemiballismus.

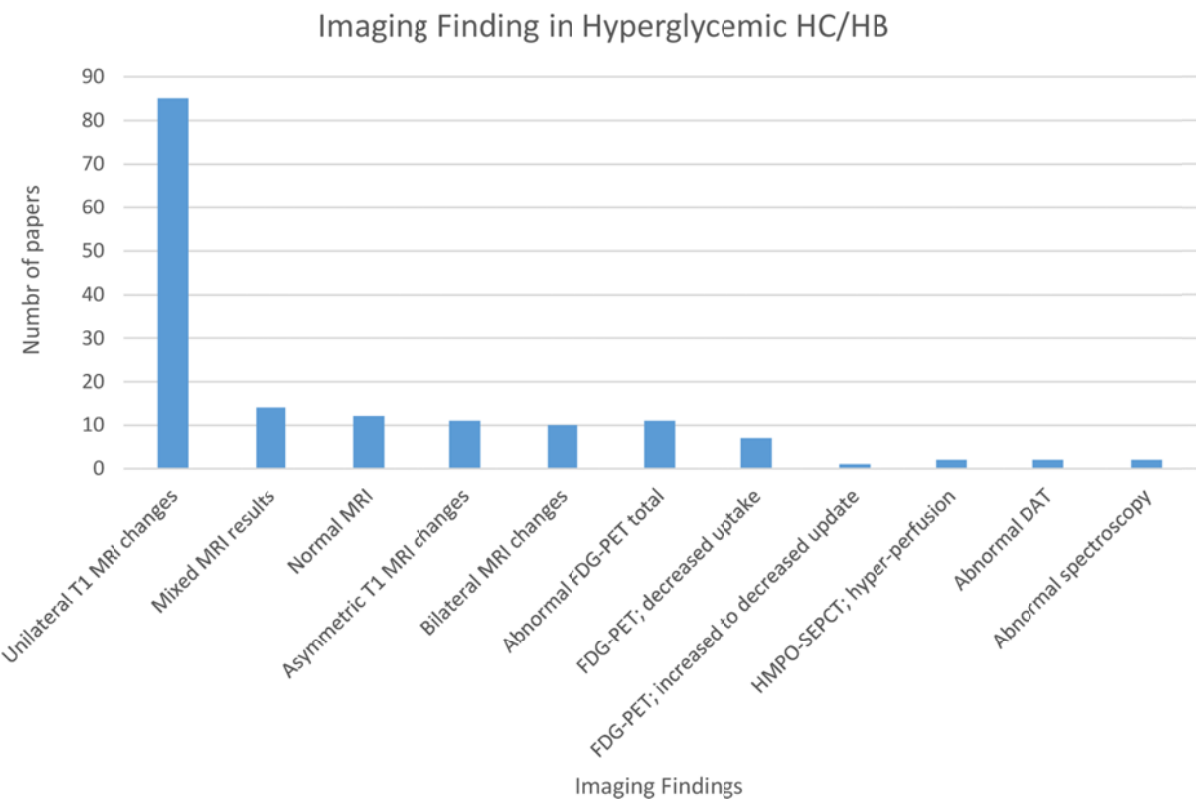

Supplement: Supplemental Figure 3. — Imaging findings in hyperglycemic hemichorea/hemibalismus. [file tohm-12-1-675-s3.pdf]
