# Supplementary material for: One Side of the Story; Clues to Etiology in Patients with Asymmetric Chorea
Source: Tremor Other Hyperkinet Mov (N Y). 2022 Jan 31;12:3. doi: 10.5334/tohm.675 (PMC8815437; doi:10.5334/tohm.675)
Supplement: Supplemental Figure 2. — Number of papers by diagnosis. [file tohm-12-1-675-s2.pdf]

Supplemental Figure 2: Number of papers by diagnosis.

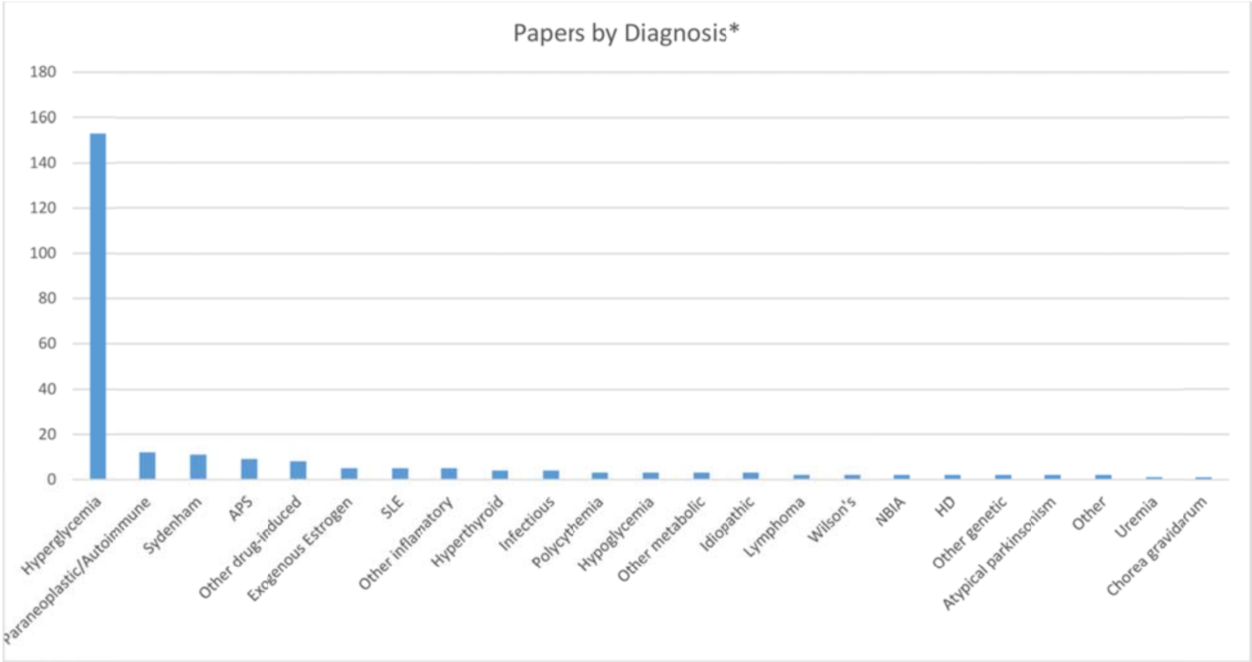

* Papers including at least one asymmetric case included. Dual diagnosis categorized under both diagnoses.
